# Non-use of Digital Health Consultations Among Swedish Elderly Living in the Countryside

**DOI:** 10.3389/fpubh.2021.588583

**Published:** 2021-09-10

**Authors:** Sara Landgren, Åsa Cajander

**Affiliations:** ^1^Department of Informatics and Media, Uppsala University, Uppsala, Sweden; ^2^Department of Information Technology, Uppsala University, Uppsala, Sweden

**Keywords:** e-health, digital health meeting services, digital healthcare, primary care, elderly, rural, countryside

## Abstract

Digital health consultations in primary care have the advantage of offering equal healthcare for people residing in the countryside. While it is gaining acceptance among young- and middle-aged people, the elderly are reluctant to use it. The aim of this study was hence to identify reasons for non-use among elderly in the countryside and describe perceived possible challenges and opportunities with digital health consultations. Semi-structured interviews were conducted with 13 persons over 65 years old residing in the Swedish countryside. There was a mistrust for services offered by private companies and their public funding, a lack of knowledge of available services, and a lack of perceived usefulness. Personal interaction and continuity was more important than time or travel conveniences, although these advantages were recognized. To prevent digital exclusion, caregivers need to offer information, encouragement, or tools for the elderly. Digital primary care also needs to offer familiarity, with continuity and personal connections.

## Introduction

An unprecedented development and innovation in e-health has resulted in the creation of new ways of delivering different kinds of healthcare digitally (1). Among the driving factors for this rapid development lie promises of increased healthcare equality, efficiency, and cost-effectiveness (2). Another potential advantage, which due to the ongoing COVID-19 pandemic, has been made notably relevant, is the possibility to deliver healthcare to those in need while protecting patients and staff from infection. Indeed, healthcare providers worldwide have been quickly changing their healthcare delivery, with digital video consultations that have been of particular interest (3, 4). This advantage is essential in protecting the most fragile population, namely those with underlying conditions and the elderly (5). Moreover, those residing in more rural areas, with limited healthcare availability nearby and long distances to specialized care, stand to benefit the most from this development (6).

With technological advances, many new digital healthcare services have emerged in Sweden in the last few years (7). Examples of these services are video and chat services used for consultation in primary care (8), (from now on referred to as *digital health consultations*). These services were initially introduced by private care providers (9, 10). In response, an increasing number of public health care providers have introduced digital services to complement their traditional offerings (11, 12). In addition, many regional councils in Sweden have already implemented, or are developing proprietary digital primary care applications or collaborating with private providers (13). The Swedish government recognizes this as a possible way to shorten waiting times, and is in the process of supporting and aiding the regions with developing these solutions together with 1,177 Vårdguiden (14).

However, the emergence of these ventures in Sweden has not been without controversy. Criticism has been raised regarding private care providers receiving financing and profiting from public funding (15). Another, perhaps more essential criticism, is that these services cater primarily to the younger, technologically competent, and healthier individuals (16). Recent numbers support this, showing that few elderly (65+ years old) use these services (8). Perhaps unsurprisingly, the elderly are often amongst the late adaptors in new technology implementations, which is frequently attributed to reasons such as “computer anxiety” and poor understanding of usefulness (17–19). This can result in “digital exclusion” amongst some elderly (6, 20). Thus, despite the elderly in Sweden having higher digital skills and using internet comparatively more than in most other countries (21, 22), they still risk digital exclusion (20). This is unfortunate, as these services offer the potential to alleviate a significant problem, excluding rural residents from access to equal healthcare. Furthermore, rural areas often lack healthcare infrastructure and access to specific specialist competence (23). The problem is exacerbated by the fact that the rural population is growing older and is generally poorer than urban populations (18, 24). The challenges above make it imperative to understand why the elderly are not using these services, particularly in rural areas. Studying this is of utmost relevance for potentially more significant societal benefit. It can offer insights into how to deliver equal healthcare, and the field of e-health and Human-Computer Interaction (HCI).

A recent Swedish study on elderly patients with chronic diseases attitudes toward different digital service tools in primary care gives us some insights on this topic; most seemed satisfied with the present primary care model and did not see a need for digital devices (25). There was also a mistrust in poorly designed IT systems and doubts about their abilities to cope with technology. However, they were somewhat curious about learning more. However, to our knowledge, no study has examined the opinions and attitudes toward digital health meeting services of elderly residing in rural areas who lack prior experience.

HCI is a broad field encompassing various topics relating to human behavior and technology, often focusing on technology users (26). Granted, this is fine for gauging and evaluating various phenomena relating to how people perceive and use technology. However, an important and often underappreciated part of HCI is the non-use; partly, as non-users can be considered as potential users, but perhaps more importantly, they harbor information on what makes them non-users. This, as non-use often, is not a passive process but instead motivated, considered, and meaningful (26). The elderly often belong to late adaptors, and digital care has particular difficulties reaching this population.

The primary focus of the research is hence to examine the thoughts and opinions of elderly persons (over 65 years old) living in Swedish urban countryside, who lack prior experience with digital health meeting services; in order to define reasons for their non-use, explore their attitude toward digital technology and opinions on traditional contacts, and identify and describe what possible challenges and opportunities for digital health meeting services that they see. The research questions addressed are:

- What factors contribute to the non-use of digital health consultations among the elderly in rural Sweden?
  ° What are the general attitudes toward digital technology?
  ° What are the opinions on traditional ways of primary care consultation?
  ° What challenges and opportunities do the elderly in rural areas see with digital health consultations?

## Background

### Elderly and Technology

Several studies have been conducted throughout the years concerning technology and the elderly. They have given us insights into the difficulties in reaching user acceptance amongst them (19, 27, 28). Consistent in many of these studies, perceived usefulness and perceived ease of use are of great importance for use intention. Perceived usefulness is lower in the elderly as they often weigh in what it takes to learn the new technology (19). However, skills in digital technology are becoming more of a need rather than a choice as technology continues to develop (29). Accordingly, one study suggests that there should be strategies for developing skills and self-efficiency among older adults (29). Another study reveals the importance of providing training tools or support services when developing new technology (30).

Other factors have been suggested as barriers causing difficulties. One recurring theme is the fear of failure or lack of confidence in using new technology, sometimes dubbed “computer anxiety” (17, 19, 31, 32). In a study about technology acceptance among elderly and young adults, older people, in general, had a higher need for assistance and encouragement than younger generations (28). This is consistent with results from another study, where elderly persons were found to perceive computers as the domain of younger generations (31). It is essential to understand what factors underlie this anxiety and even more to understand what factors are significant in affecting their attitude and usage to tackle them and effectively design services that are likely to be used by the elderly. There is likely more at play than computer anxiety. Although, while women are less frequent than men in their use of digital technology, they show more interest in health information online (33).

#### Elderly and Digital Services

It is of particular interest to note studies examining the elderly's perception of other online public services such as e-banking and e-government services. These services are also intended to be aimed toward the population as a whole. While not directly comparable, they could offer valuable lessons from hurdles and potential design considerations to make the services appeal to elderly populations. In recent years, sectors such as banking have moved toward more or less total digitalization of their services in Sweden (34, 35). Many elderly are now adapting to these developments, with 60 % of the population older than 65 years having an electronic identification (called BankID) in 2019 compared to only 11% in 2015 (34–36).

Significant factors shown to influence the elderly's attitudes and intention to use services such as online banking negatively include lack of personal contact, self-efficacy, technology discomfort, and personal attributes such as age, gender, health, and poor digital skills (27, 37). Perhaps unsurprisingly, perception of safety and trust is also an essential important factor affecting acceptance in both self-service banking technologies and e-government services (37, 38). Supposedly, all of these factors could affect their perception of e-health. It is similar in many ways to other public services.

#### Elderly and E-Health Services

Previous national and international studies have provided insights into some barriers preventing the elderly from adapting to e-health services, but further research is warranted (31, 32, 39). Consistent with these studies on user acceptance, the elderly use e-health services less frequently than young people (8, 40). In addition, low confidence is more common amongst the elderly than young people when using e-health services (17). Confidence and digital technology skills are recurring themes in the literature on e-health, with the elderly often doubting their skills when facing new technologies (41). Nonetheless, low confidence and computer anxiety aside, the elderly still use the Internet more to research health information than young people (17). Like the previous mentioned studies on electronic government services and online banking, trust in technology is essential for the elderly when using e-health services (41, 42).

In a recent literature review on older people and e-health, several barriers were identified to explain the non-use of e-health services; amongst these were impaired sensory, motor, and cognitive performance (41). These are essential aspects to consider, as many elderly persons might perceive digital healthcare solutions as positive but may also be too weak or sick to understand and correctly use them (43). This is suggested in a study about video consultations in primary care, where hearing impairment was a problem for some elderly (43). Thus, the users cognitive and health status should be considered in the design process, more so in e-health applications than most other services. Failing to address this may result in creating an even bigger digital and social exclusion (6). Indeed, it may be necessary to use specific strategies to undertake the distinct needs of people in various age groups, as suggested in a recent study (17).

### Digital Health Consultations

There are currently numerous digital services in Sweden that can be referred to as e-health services; for instance, access to medical records and information online, online booking systems for healthcare, and digital consultations (7). However, in this paper, digital health consultations will only refer to the video and chat consultations used in primary care offered by private and public care providers.

The development of digital health consultations in Sweden has been increasing rapidly in the last few years (10). Being the first to implement digital health consultations, private care providers are unsurprisingly behind the majority of this increase, with the regions now attempting to catch up (10, 14). When private care providers started to offer these services, they became part of Sweden's public health offerings, which made them accessible to the whole population and granted access to public financing (10). However, as the number of these services increased rapidly, costs spiraled for some regions, and the “exploitative” business models of private care providers were publicly criticized through various media outlets (44, 45). As a result, the Swedish government formulated guidelines stating requirements that digital health consultations need to fulfill to receive compensation and limitations on charging (10).

Both chat and video consultations are offered through both mobile applications and web platforms. These services are commonly used when patients have questions about their condition, follow-ups after medical ordinations, and general consultations and diagnosis (46, 47). Although different providers utilize different ways to access their services, commonly, the patient signs in to the application or web platform with a mobile electronic ID (BankID), then answers questions and describes their issue before receiving a video call back in the application (48).

One study found that young and old patients familiar with text messaging were equally willing to adapt to the technology for communicating in text with their doctor (49). This is interesting since a recent Swedish study published in collaboration with SKL (Sweden's Municipalities and Counties) shows that the usage of digital health consultations differs significantly depending on age (8). The authors examined how inhabitants used digital health meeting services in the Swedish county of Jönköping between September 2017 and January 2019. They found that usage was highest among children, that usage continues to be high amongst young adults but already declines to half of the numbers for the population 31–50 years old. When looking at 65+ years olds, their usage was by far the lowest with four meetings per 1,000 inhabitants (compared to 29 on average for the total population) despite representing almost a quarter of the population. While these numbers are from one county in Sweden and some local variations are likely to exist, the results should be transferable to represent the current situation in Sweden as a whole. These statistics bring up an exciting inconsistency; if attitudes might be similar between young and elderly, but usage differs significantly, what other aspects impact their usage more than their attitudes?

#### E-Health and Rural Areas

There is no general definition for rural areas, and they vary in literature and different countries. However, commonly an area is defined as rural depending on population and geography (33). In this context, it is shortly defined as any sparsely populated settlement outside of more urban cities.

A recent study about today's e-health solutions in Sweden has brought up the potential creation of “digital health landscapes”, where geographical distances will not be of concern when delivering healthcare to patients (6). Possible positive outcomes discussed in the study were that people living in sparsely populated areas in Sweden could access equal healthcare as people living in urban areas (6). This should not be underestimated, as Swedens vast and sparsely populated geography complicates and increases the cost of delivering equal healthcare to all citizens (6). In addition, attracting competent medical staff to rural areas is often difficult and expensive (33).

While these solutions seem positive for elderly and rural residents, insights from a government study in Sweden tell us that younger people consume more e-health services than the elderly and that residents in socio-economically vital areas consume more than people living in rural areas and countryside's (18). Moreover, countryside residents' health tends to be lower in general than people residing in cities (18). However, it is conceivable that some of these services need some time on solid markets to get a higher spread in usage, with young people often being among the “early adopters” (18), meaning they are the first to use new services.

Literature also shows that video consultations might not always be the preferred method of choice for people in rural areas, with issues in image quality being of great concern (43). In addition, the same literature brought up concerns about the ability of the physician to properly understand the patients' problems when not meeting face-to-face. In another study, the authors found similar results; people in rural areas saw the distance to care as an essential factor for accessibility, but were unsure if video consultation was a suitable solution (50). The main reasons were uncertainty about what problems these consultations were ideal for and what consequences digitalization could have on existing rural primary care.

Current findings on rural aspects of e-health are indeed interesting. However, few of these focus exclusively on the elderly and digital health consultations. Moreover, no study focuses solely on these aspects in the elderly with no prior experience to the best of our knowledge. As it currently stands, the elderly who have used these consultation services belong to a minority population. Focusing on the higher number of elderly who have not used digital health consultations would arguably provide even more valuable insights, especially what factors prevent them from trying. Further, with Sweden's vast and sparsely populated geography, it is of particular interest to study non-users in the countryside. They are often restricted in their access to healthcare and potentially stand to gain the most with properly implemented digital healthcare.

### Summary of Previous Literature

The elderly have been shown to have lower confidence and doubting their skills in technology than young people. Moreover, they were shown to have a higher need for encouragement than younger individuals. Despite these hurdles, skills in digital technologies were shown to be high among the elderly in Sweden, with many frequently using e-services such as e-banking today. Factors of great importance affecting elderly person attitudes toward technology and e-services were personal contact, perceived usefulness, and perceived safety and trust. When it comes to the distances (regarding rural areas), it has been recognized as an essential factor for accessibility. However, in the literature on the elderly with experience of video consultations, it was unclear whether it always is a suitable solution. There is also a lack of research on non-users.

## Methodology

### Data Collection

The research question was explored qualitatively through semi-structured interviews with 13 subjects belonging to the population of concern (65+-year-old individuals). It was part of a master's thesis in Human-Computer Interaction during spring 2020. The present study carries both a deductive and an inductive approach. Deductive in that the interview questions were formulated based on previous literature of relevance and inductive in that there were no strict predefined themes for the analysis, with new themes identified during result analysis (51).

Based on the research question and previous studies, an interview guide was created to formulate the interview questions. Before the primary interviews, a pilot study was conducted on three individuals (including a physician) to evaluate the questions, estimate time requirements, and prevent too complicated and/or unclear questions. Following this feedback some questions were reformulated to refrain from gathering personal medical information, redundant questions were removed and some sections were reordered for clarity. To avoid influencing the participants, no specific background information on digital health consultations was provided prior to, or during the interviews.

#### Sampling

A purposive strategy was used for sampling. The target population was people over 65 years old who had not used digital health consultations before and lived in the countryside of Jämtland in northern Sweden. The countryside is defined as settlements with less than 2,000 inhabitants and with 5-45 minutes of travel time by car to the nearest city. Qualitative studies with semi-structured interviews are recommended to include a sample size of 5–25 (52). This study aimed to interview at least 12 people, preferably with an equal distribution of male and female participants to reach saturation.

The participants were recruited at meetings arranged for senior citizens in Swedish villages; they were presented with a written description of the study. They could express their interest in a form, where they also confirmed that they were over 65 years old and had not tried digital health consultations before. Further information was later provided to the interested, including policies on the handling of personal information. Written consent was collected from participants before all face-to-face interviews. During the phone interviews, a verbal statement was recorded and saved.

#### Semi-structured Interviews

A total of 13 participants were interviewed, seven of whom were female and six male. The mean age of all participants was 73, 6 years, with the female participants being slightly older than the male (mean age 74, 6 compared to 72, 5). The oldest woman was 82 years old, and the oldest male was 76. Education levels varied from secondary school and lower (9–12 completed grades) to university, with most participants having vocational education or higher. Furthermore, the entire sample population lived in countryside settlements, some of which did not offer any healthcare services, while others did. All interviews were conducted in the participants' native language (Swedish) to make it convenient for them and not miss any valuable information.

#### Ethical Considerations for Data Collection

The national recommendations for ethical approval have been met, and all international ethical guidelines have been followed. The interview questions were formulated to be as objective and unbiased as possible, avoiding leading questions and positive or negative connotations. To achieve this, an interview guide was constructed based on themes identified in previous literature. To further avoid irrelevant and or inappropriate questions, a pilot interview was conducted.

This study intended to gather opinions on why elderly persons residing in the countryside have not tried digital health consultations. No medical information or background was collected. The study's intention was carefully explained to the participants, including information on their right to withdraw from participation at any time, and that personal information would not be accessible to anyone besides the author of this study. Data processing and storage conforms to GDPR (ensuring adequate protection of collected personal information) (53).

### Data Analysis

#### Thematic Analysis

A thematic analysis was conducted following Braun and Clarke (54): familiarizing with the data by transcribing and reading it through, generating initial codes, searching for themes among the codes, reviewing the themes, defining the themes, and finally selecting extract examples. All coding was performed using a qualitative analysis tool. The predefined overlaying categories based on previous studies were: *skills, attitude*, and *distance*. The themes that emerged from the thematic analysis were: *concerns and skepticism, personal interaction and continuity, knowledge*, and *perceived opportunities*. For an overview of the themes see Table 1.

**Table 1:** Three predefined themes (skills, attitude and distance) based on previous literature.

**Theme**	**Description of theme**
Skills	Prior and current knowledge and usage of technology and e-services.
Attitude	Attitudes toward technology, digitalization in sociaty and digital healthcare.
Distance	Distance to the nearest helathcare center and availability of healthcare on the countryside.
Concerncs and skepticism	Concerns on different aspects of digital technology, the digitalization of healthcare and digital health consultations.
Personcal interaction and continuity	Views on patient-doctor interaction and the importance of continuity in healthcare.
Knowledge	Knowledge about digital health consultations; availability, how it works and when it can be used.
Perceived opportunities	Opportunities that the elderly see with digital health consultations.

*Following the interviews, a thematic analysis identified (concerns and skepticism, personal interaction and continuity, knowledge and perceived opportunities). All seven themes were used in the final data analysis*.

After the analysis, the themes were grouped in a meaningful way to answer the research questions. All quotes presented in the results were initially Swedish but have been translated to English and made more readable.

## Result

### Attitudes Toward Digital Technology

Almost all participants were using digital services daily. The two that did not were both amongst the oldest participants (both over 80 years old). These two were the only participants who did not consider themselves confident in using digital technologies such as computers, smartphones, or applications. All remaining participants considered themselves as either “pretty confident” (six participants) or “confident” (five participants).

All participants were using smartphones or computers daily. Moreover, all were familiar with sending text messages using their phone. Overall, the participants were proficient in digital technologies (such as smartphones, computers, applications), with all but two using multiple services daily. The two remaining participants were again the oldest in the study (both over 80 years old); however, they have access to technology, with one of them owning and using a smartphone and the other a computer. The 11 participants who used digital services daily commonly used social networking applications to contact friends and family. All but one used online banking services. The remaining participant relied on help from a younger relative for such errands.

Overall, most participants had positive attitudes toward digital services, describing them as valuable and time-saving. One participant describes the convenience: “*It is really efficient, I can even pay an invoice immediately when I get it. And it gets paid correctly. And I can do transfers on my bank between the accounts by myself, and that is really convenient.”*

### Opinions on Traditional Primary Care and Consultations

All participants traveled by car (driving themselves) to their health center when heading for an appointment. Furthermore, eight participants had over 10 km to their health center, and considered themselves dependent on their car for attending appointments. When discussing the possibilities of using public transportation, most mentioned poor public transport services that make it challenging to find a bus that would match the appointment time.

All the participants, except for one, primarily contacted their health centers via phone calls for booking appointments, with the remaining participant using the online service 1,177 Vårdguiden. The majority of the participants positively mentioned how easy it was to get an appointment at their health center. Further, some of them talked fondly of a voicemail feature that their health centers offered; when the phone line was experiencing prolonged waiting times, you could provide your personal phone number and have someone from the health center call you back at a later time. One participant explains: “*I call the health center on their phone number. Then you have to wait, but if you say your personal number, they will call you. And that works really well.”*

Most participants had experience in using the online health service on 1,177 Vårdguiden for looking up their test results, book appointments, or renew their prescriptions. One of the participants expressed that he prefers to book his appointments online: “*Nowadays I don't call anymore, instead I am using Vårdguiden. Or 1,177… Or what it is called.”*

Besides contacting the health center via phone or Vårdguiden 1,177, two participants had also booked appointments or requested contact with their physician through written notes, for example, when conducting blood pressure self-tests at the healthcare facility. One of them describes it: “*... and then you write on a note which pressure you have, and then I usually write there that I want to come in contact with my doctor.”*

Some participants stated that they were connected to a personal family physician at their health center and recognized that this was of great importance. They considered it convenient and suitable for continuity without repeating their medical history with each doctor's visit. It was also perceived as more safe and trustful that the doctor personally knew them as patients. Two participants revealed that they previously had changed to health centers located further away from their residence to keep their family physicians who had changed their workplace.

Overall most participants were satisfied with the primary care, although some raised a few points and complaints. One common complaint was poor follow-up routines. Most participants mentioned that following appointments, responsibility regarding follow-ups were often placed on themselves. This included medication follow-up and prescription renewals. In instances where the health centers did reach out to them, the communication was conducted through written letters or phone calls. One participant expressed himself positively about the transition to increased digital communication: “*They send letters, which I think is a little unnecessary today. But, I think they are changing a little and sending calls or follow-ups digitally, and that is way better.”*

### Reasons for Non-use and Current Challenges

No participants included in this study had any prior experience with digital health consultations. To delineate possible reasons for this, their knowledge of and attitudes toward these types of consultations were explored.

Regarding knowledge of available digital health consultations services, most participants mentioned that they had seen ads for private companies offering this. Some responded that they knew nothing about how it works. Two said they imagined it being similar to other text messaging or video conferencing applications. No participant was aware of publicly financed digital healthcare. No one had any knowledge if their health center offered such a service. The participants, in general, did not know much about these services, how they work, and who is providing them. Thus, they were described to them in more detail.

Most participants were content with the current status of the primary healthcare system, and a few noted that they had not felt the need for using a digital alternative. Some participants stated that they had not engaged in the development of digital health consultations. One of them felt he lacked the energy and motivation to do so. Some participants also believed digital health consultations were a generational matter, not designed for the elderly population. One participant expressed: “*For the people who are working in this digital world, this is a meeting for them [...] So I believe it is really a generation thing.”*

The participants shared some concerns and fears for the concept of digital health consultations, either about themselves or based on thoughts about other elderly. A commonly raised concern was that older people have difficulties using various digital technologies. Some participants believe that many elderly are afraid of these types of developments. A few participants raised concerns that users need to be technologically knowledgeable to use these kinds of healthcare services. Some were afraid that they would not be able to keep up and use it when getting older. Two mentioned that relatives might be able to help with potential technical difficulties; one of them said that she was concerned that the elderly might get left behind and excluded in the digitization of healthcare. She also believed that the elderly should receive financial support to purchase a computer and be offered education on digital technologies. The participant stated she would be interested in such an education. Lastly, a few participants were concerned that the increase in digitization of healthcare might lead to reduced employment in traditional healthcare.

One relatively common concern was the physician's feasibility to properly examine and derive a diagnosis without meeting the patient in real life. Most participants raised this concern, where one of them said: “*It feels like it can be a little difficult to get a correct diagnosis… Just with a camera or an app… Even though you sit there and talk. It would feel safer to sit and talk with a doctor eye to eye”*.

Some participants displayed various degrees of skepticism toward digital health consultations since they felt that these types of meetings were too impersonal. One participant considered himself too conservative.

By far, the most common reason for skepticism toward digital health consultations was financing. Many participants were very concerned about private care providers profiting from these consultations by charging the region. They had either heard from others or read themselves from news sources that private care providers were exploiting the reimbursement system for these consultations and wished to take a firm stance away from this; “*You get a notification [from a news service] on your phone that… ‘Don't use that; it costs this and that'… So I have thought that ‘no, this is something I probably will never use', because I don't want it to cost as much as it says [for the region].”*

Most participants that were skeptical about private care providers said that they could imagine themselves using this type of service if it was publicly controlled and preferably connected to their current health center. The remaining participant, while recognizing this type of solution as something positive, was still reluctant to consider trying it themselves as personal contact was too essential to overlook: “*It should be governed by public health care institutions, that there are no private companies. Then I think it is positive, but I will never use it [...] because I want to have a personal meeting, I think it is really important.”*

### Perceived Opportunities and Desires

Despite the concerns and skepticism outlined in the previous sections, the general attitude toward digital health consultations was positive. Given that their primary concern of private care providers profiting from digital health consultations was addressed, or public alternatives were made more broadly available (mainly, if the participants' own health centers would implement digital consultation features), most participants could see benefits and consider utilizing digital consultations. The majority of the participants responded that they believe digital health consultations are, or can be positive. All of them saw convenience and the potential to save time as the main advantages. In addition, one participant identified the potential benefit of a lessened risk of infection from other patients when conducting digital consultations.

As previously mentioned, all participants were reliant on their vehicle for transportation to health consultations; most participants thus recognized digital health consultations having the potential of being particularly beneficial for people residing in more isolated rural areas. For some, the ordeal of traveling to their health center or hospital for a short visit could take the whole day. Some pointed out that sufficient internet connectivity is crucial to benefit from digital health consultations but is not always available. A few also pointed out and recognized that while they may be independent now, their situation might change in the future, preventing them from driving, which made digital health consultations seem more alluring to them in the future.

Continuity was essential to the participants, most of whom preferred having repeated contacts with the same physician. They said they feel safer when the doctor knows them. However, opinions differed significantly between the participants. At the same time, two claimed that they would go as far as to change their health center to stay with the same physician. Others felt that continuity was not always necessary (particularly in more urgent situations). Many participants could see themselves using digital health consultations, but having their own family doctor and health center was a decisive factor for many and preferred amongst most others.

The importance of personal contact was a common theme throughout the interviews. Most participants reported that personal contact (in various forms and degrees) is essential to them. For example, one participant reported favoring waiting in a physical waiting room over waiting on the phone, mainly because of the human contact and interaction. Another participant did not mind phone meetings, but did not care for digital booking solutions through 1,177 Vårdguiden due to the lack of personal interaction. Some participants said eye contact was of particular importance for meetings in a healthcare setting. Reasons for this, amongst others, were that it allows for non-verbal communication of empathy and compassion and can signal mental or health status. Related to this, one of the participants brought up the importance of a pleasant treatment when in contact with the healthcare provider, implying that this is of greater importance than the mode of contact: “*If you only get a friendly response, then it doesn't matter if I see the person or not. If I get properly cared for over the phone, and they understand what I mean, then it is totally fine I think”*.

If they were to use digital health consultations in the future, most participants stated that they would prefer video calls over chat. The main reason for this being that it feels more personal and allowing for eye contact with the healthcare worker. Moreover, several participants saw that this would also aid in the physical examination. A few participants reported that they would prefer using a computer over a smartphone in such a meeting. The primary reason for this was a perceived lack of proficiency in using smartphones, mainly in slow typing speeds. In addition, the participants attributed to the small size on smartphone keyboards. The participants who were open to chat meetings with their health care provider could only consider this on the computer. A few participants preferred using a smartphone application, mainly as this was perceived as more flexible than using the computer.

Some participants brought up requests and desires for the functionality of a digital healthcare application. All were in some form related to the contact with the physician; a few wished to choose whether to book a digital meeting with their family physician or reach any doctor immediately for an urgent consultation. Another participant wished for an easy way to contact the consulting physician afterward if any questions arose. The last participant wished that digital services would offer follow-ups by the same healthcare personnel, similar to how it works currently in physical health centers.

## Discussion

This study aimed to identify reasons for the non-use of digital health consultations among the elderly in the countryside, and describe their perceived possible challenges and opportunities. As a result, several interesting insights were gathered by interviewing 13 persons belonging to this population in a semi-structured and qualitative manner. While some previous findings were confirmed, others were of lesser importance in this population, and some new aspects were discovered.

Indeed, the most commonly stated reason for the non-use of digital health consultations was the matter of its funding. This view coincides with the primary general critique that private digital healthcare has received in Sweden (44, 45). This suggests that the same factors affect both the opinions of younger and older populations in some ways. Granted, this opinion may have less of a deterring effect on younger individuals. Nevertheless, most interviewed participants were uneasy supporting private healthcare providers because their financing was sourced from public healthcare funds. An inherent limitation of the current study is its somewhat limited sampling heterogeneity, as all participants originated from the same county and shared many interests and views. This topic needs to be further investigated since opinions could vary in different regions and these findings. Moreover, healthcare in Sweden has recently been more or less exclusively public. The recent upswing of private care providers in Sweden has been perceived as daunting by many (15). Thus, this reason for non-use likely would not translate well compared to countries where private and public healthcare coexists, or is dominated by private providers. Besides this, the other main reason for skepticism and non-use was concerns regarding personal contact and interaction.

Overall, personal contact with the healthcare provider and treatment was deemed as more critical than conveniences. For example, multiple participants were prepared to travel a further distance for a physical meeting with someone they were more comfortable with. With that said, most participants were also open to putting aside their reservations and trying digital health consultations, mainly if they were possible to conduct with their current physician. Literature has shown that a lack of personal contact negatively influences the acceptance of e-services by the elderly (37). Similarly, the participants in this study thought that personal contact was more valuable than the potential saved time and efficiency it could offer.

Somewhat related, the aspect of continuity was decisive for many participants. Here again, they expressed a desire to consult their physician whom they trust, who is familiar with their medical history, and who knows them on a more personal level. The participants perceived private care providers to have limited possibilities to offer such services. If their health centers would offer digital consultations, this barrier of perceived impersonality and mistrust could be more easily overcome. Here, education and knowledge of current offerings also come into perspective. Many public health centers are already offering some digital services. However, private care providers where the first to reach the market and are overrepresented in marketing, overshadowing and obscuring the perception of what is available.

Another substantial reason for non-use among the participants was the lack of perceived usefulness. They were satisfied with how their contact with primary care today and failed to see any potential improvements digital healthcare could bring. Of course, the time required for learning a new technology is a factor for the perception of usefulness (19). However, in the current study population, this was generally not the case. Instead, it was the perceived lack of personal contact and lack of safety and trust (especially for private care providers) that affected their opinions on digital healthcare. For instance, most participants could see numerous benefits that digital healthcare could bring, such as increased practicality, saved time, improved healthcare coverage (especially for more isolated rural/countryside residents), increased flexibility, and decreased risk of infections. Nonetheless, the perceived lack of sufficient personal contact and mistrust for private care providers outweighed most practical benefits. These are exciting findings, as one might expect that aging rural residents with poor public transportation availability and a dependence on personal transportation by car would value these aspects higher and consider digital services more valuable.

Literature showed that trust in technology's integrity and safety is essential (42), which was not considered a concern among this study population. Most participants believed it was essential to feeling that the healthcare workers in digital healthcare were competent, caring, had access to their medical records, and genuinely interested in helping. Indeed, emotional investment and good contact with the treating physician appear to be decisive factors for elderly persons' commitment to use digital health consultations. One hurdle might be the overrepresentation of private ventures, as many participants expressed mistrust for these. So, if the patients could contact their health center and physician that they trust and recognize, many participants were open to trying digital health consultations. As more public caregivers are implementing digital services, it will be interesting to see if this has an impact on their usage. The importance of personal contact and emotional investment from the care provider reflects the participants' preferences for digital contacts. A vast majority prefer video calls over chat.

Multiple participants expressed concerns about the healthcare workers' abilities to properly examine and diagnose the patient by digital means, which concurs with what previous literature found when they studied video consultations in primary care (43). Again this partially stems from preconceptions and poor understanding of what types of consultations can be conducted through video calls and chat.

Previous literature suggests computer anxiety to be one of the main reasons for elderly's reluctance to adapt to new technologies (17, 19, 31, 32). Somewhat surprisingly, this was barely an issue at all with the study population. Almost all participants were very comfortable with modern technologies. The two oldest participants (who were both in their eighties) did express some anxiety but were still optimistic about the idea and open to learning more and trying digital health consultations if only some assistance were offered at first.

As the participants have sufficient skills in using digital technology, and in a broader sense, are favorable to increased digitization of public healthcare, a reasonable first step could be to increase their knowledge on and comfort with digital health consultations and services. This process needs to be gradual to prevent causing exclusion and paralleled with ample information and education. Essential to consider here is also which type of communication channels are offered to elderly patients. The participants perceived smartphones as small and expressed difficulties with writing quickly, such as in a chat. Video calls could thus be a more preferred mode to contact healthcare digitally. Although the elderly in Sweden have high digital skills (22), as was the case for the participants in this study, it is conceivable that attitudes and literacy could differ in a broader study.

At the time of writing, the global COVID-19 pandemic is ongoing, bringing numerous consequences and altered recommendations, particularly for the elderly (5). The interviews in this study were conducted before the mass outbreak in Sweden. Since then, persons above the age of 70 were recommended to self-isolate as they were considered risk groups (5). However, even before the pandemic outbreak, some participants recognized the benefits of a lessened risk of infection with digital health consultations. Even without ongoing pandemics, the yearly influenza outbreaks are coupled with an increased death rate among the elderly (55).

Earlier findings on technology acceptance have suggested that the elderly are in greater need of encouragement and assistance (28). While this might seem obvious, there is still much that can be done. For instance, there was a general lack of knowledge and information on digital care amongst the study population. General news media was their primary source of information. With digital healthcare expected to increase in prevalence (14, 56), explicit measures must be taken to include the elderly in this expansion, seeing that it is unlikely that they will spontaneously find the motivation to adapt to this new and foreign concept of care.

The study population pointed out a lack of follow-ups in the current primary care. This could be one opportunity to integrate the elderly more into the digitization of healthcare. For example, follow-ups that do not require extensive physical examination could be offered through a video call. These types of meetings could allow the patients to familiarize themselves with the concept of digital health consultations and intuitively learn its possibilities and limitations without the personal and emotional stress of a regular, more severe, and urgent visit. While there is no guarantee that this would affect their behavior, based on the insights gathered from the current study population it seems to be an appropriate first step that could be interesting to evaluate on a smaller population.

Efforts must be made to adapt digital care not only to the potential needs and limitations of the elderly but also to prevent exclusion. No matter how digitally literate and confident, the elderly cannot be expected to integrate and come to digital care themselves - digital care must come to them by being inclusively structured with their preferences and opinions in mind. In addition, special considerations must be made for the aging populations in rural areas, with the potential limitations such as internet connectivity - as was brought up by some participants, despite themselves not suffering from that problem. This is perhaps the issue; today's elderly are technologically competent and open to digitalization. Their reasons for non-use of digital health consultations appear to match the general issues and critiques of the population. Howver, there is an evident lack of information on availability and options and encouragement from public care providers.

While it is of particular importance in today's global situation to protect our elderly, we should feel motivated to do so at all times. Thus, care providers could take more responsibility and encourage digital care if they wish to transition into more digitilized healthcare. With the elderly in the present study expressing trust in their current health centers and physicians, public care providers should acknowledge this trust when implementing digital services, not doing it carelessly, and providing ample support and knowledge. With good information and methodological introduction, the elderly can become familiar with digital health consultations and can accept it, at least based on the opinions and insights gathered from the current study population.

## Limitations

As both the field of e-health and the technological competence amongst the elderly rapidly advances, early studies lose their relevance and ability to be generalized in current times. Moreover, different populations, access to the internet, personal computers, and smart devices should be kept in mind when studying international literature. In international comparisons, Sweden is frequently at the forefront regarding internet usage and digital skills amongst the elderly (21, 22).

This study does not fully capture all aspects of digital health consultations as the participants lack prior experience with such services. Furthermore, the elderly (65+ years old) is a vastly heterogeneous population. While the needs of some individuals might correlate with those of younger populations, but as age increases, so does the relative proportion of people with different healthcare requirements. For example, this study population was dependent on driving but could do so independently. Moreover, the definition of countryside can vary greatly. It would thus be interesting to look more into the opinions of the elderly who are more dependent on public transport or help from others and those residing in more isolated rural areas. Finally, as mentioned, the opinions on private care providers might also be specific to this study population and primarily limited to Sweden.

## Conclusion

This study aimed to identify reasons for non-use of digital health consultations among the elderly in the countryside and describe their perceived possible challenges and opportunities. Based on the analysis of 13 qualitative interviews, some conclusions can be drawn.

The main reasons for non-use were: (1) concerns regarding the funding of digital health consultation services and mistrust for private care providers, (2) a lack of knowledge of available services, both from their health center, public providers, and on what types of consultations were possible, and (3) a lack of perceived usefulness as they felt satisfied with their current form of primary care and were unphased by current limitations such as distance.

Regarding challenges, personal interaction and continuity were deemed more important than potential conveniences. There was also a general skepticism in the platform's capabilities of allowing the physician to conduct a proper examination or recognize their problems. Furthermore, there was a fear of increased complexity that could result in future exclusion provided that they do not cope with the technical development.

The participants did recognize numerous opportunities; most were avid users of digital technologies and services and were open to trying digital health consultations given that their current healthcare provider offered it. They were open to the concepts of both computer and phone application based meetings, with a general preference toward video consultations as they offer to preserve the feeling of personal interaction that they value. Computers were most often preferred over smartphone given the larger keyboard and screen size. The participants could also recognize the potential benefits of digital health consultations, such as increased convenience, time-saving and lessened risk of infection.

### Future Work

Future research on a broader population of non-users, with a higher inclusion of very isolated rural residents and persons being more dependent on public transportation or the help of others, would be warranted to expand on the findings. Moreover, research on rural areas following the impact of the ongoing COVID-19 pandemic that has brought on new recommendations for the elderly and rapidly increased public healthcare digitization will be highly relevant.

## Data Availability Statement

The raw data supporting the conclusions of this article will be made available by the authors, without undue reservation.

## Ethics Statement

Ethical review and approval was not required for the study on human participants in accordance with the local legislation and institutional requirements. The patients/participants provided their written informed consent to participate in this study.

## Author Contributions

SL and ÅC designed the study. SL conducted the interviews, collected and analyzed the data. SL wrote the manuscript with support from ÅC. Both authors reviewed and approved of the final submission.

## Conflict of Interest

The authors declare that the research was conducted in the absence of any commercial or financial relationships that could be construed as a potential conflict of interest.

## Publisher's Note

All claims expressed in this article are solely those of the authors and do not necessarily represent those of their affiliated organizations, or those of the publisher, the editors and the reviewers. Any product that may be evaluated in this article, or claim that may be made by its manufacturer, is not guaranteed or endorsed by the publisher.
